# Self-medication among general population in the European Union: prevalence and associated factors

**DOI:** 10.1007/s10654-024-01153-1

**Published:** 2024-09-18

**Authors:** Spencer Yeamans, Ángel Gil-de-Miguel, Valentín Hernández-Barrera, Pilar Carrasco-Garrido

**Affiliations:** Department of Medical Specialties and Public Health, Preventative Medicine and Public Health Area, Universidad Rey Juan Carlos, Alcorcon, Madrid Spain

**Keywords:** Self-medication, Over-the-counter, European Union, Europe, European Health Interview Survey, EHIS

## Abstract

Self-medication (SM) forms an important part of public health strategy. Nonetheless, little research has been performed to understand the current state of self-medication in the European Union (EU). Utilizing data from the third wave of the European Health Interview Surveys, this study finds an estimated SM prevalence of 34.3% in the EU (95%CI = 34.1-34.5%; *n* = 255,758). SM prevalence, as well as SM prevalence inequality between men and women, varies substantially between EU member countries. Via multivariable analysis, we also identify a number of variables associated with SM, most notably the substantial impact of health systems on SM behavior (Adjusted Odds Ratio [AOR] = 4.00; 95% Confidence Interval [95%CI] = 3.81–4.21). Several demographics are also associated with greater SM prevalence, including those aged 25–44 (versus ages 75+: AOR = 1.21; 95%CI = 1.12–1.31), women (AOR = 1.74; 95%CI = 1.68–1.81), immigrants born in other EU states (AOR = 1.16; 95%CI = 1.04–1.30), those with higher education (AOR = 1.83; 95%CI = 1.60–2.09), and urban dwellers (AOR = 1.14; 95%CI = 1.04–1.30). Additionally, long-standing health problems (AOR = 1.39; 95%CI = 1.33–1.45), visits to doctors (both general practitioners and specialists) (AOR = 1.21, 95%CIs = 1.15–1.26, 1.17–1.26), and unmet needs for health care due to waiting lists (AOR = 1.38; 95%CI = 1.23–1.55) or inability to afford medical examinations/treatment (AOR = 1.27; 95%CI = 1.12–1.42) serve as conditioners for SM. We also find that smoking (AOR = 1.05; 95%CI = 1.01–1.10), vaping (AOR = 1.19; 95%CI = 1.06–1.32), drinking alcohol (AOR = 1.23; 95%CI = 1.19–1.28), and higher levels of physical activity (AOR = 1.27; 95%CI = 1.22–1.32) are factors associated with SM. Analysis of these variables reveals that though women self-medicate more than men, the patterns that govern their consumption are similar.

**Supplementary Information:**

The online version contains supplementary material available at 10.1007/s10654-024-01153-1.

## Introduction

The pervasive medicalization of our society, marked by excessive diagnoses and prescriptions, has given rise to high medication use, a public health problem [1]. However, the upsurge in medication use stems not only from inappropriate prescription writing by health care professionals [2], but also from considerable increases in self-medication (SM) [3, 4]. It has been reported that 85% of patients in Czechia attempt self-treatment before consulting a health care professional [5] and less than 20% of Australian patients reported often following dosage, frequency, and duration directions for non-prescription medicines [6]. Little consensus exists in the literature defining SM, though for the purposes of this study, it can be defined as medicines, herbal medicines, homeopathic medicines, or dietary supplements (such as vitamins, minerals or tonics) used at the respondent’s own initiative [7]. SM prevalence rates range from 11.2 to 93.7% worldwide [8], reflecting the disparate findings in research on SM.

Several factors contribute to the frequency of SM. Inaccessibility of health care due to long waiting times [9, 10], high costs, and long distances [11–13], as well as potential time [14] and cost savings, often compel individuals to turn to SM. Additional motivators are the need for immediate care (such as severe pain), the perception of current symptoms and illnesses as mild and not serious [15, 16], and the lack of recognition of symptoms by a doctor [17]. SM is also driven by prior negative experiences in health care, prior positive experiences with SM, or self-diagnosis and management of symptoms and illnesses, often which are chronic or have been previously experienced [18]. The promotion of general well-being, enhancement of cognitive and physical performance, and aesthetic and recreational purposes are other impetuses for SM. This practice is further fueled by increasing patient knowledge stemming from recommendations from non-medical professionals, access to information via the internet, and the promotion of healthy lifestyles and medicines in media and advertising. This is coupled with greater availability of medicinal substances such as OTC medications [19].

Undoubtedly, appropriate SM has numerous benefits, including empowering individuals to be responsible for their health and engage in active prophylaxis. Self-management programs and interventions are associated with positive health outcomes [20]. This practice also has the potential to boost patient quality of life through time and cost savings, and alleviation of physical discomfort for both chronic and acute conditions [21]. In turn, strain on health systems is reduced, which improves the quality and scope of treatment for those with serious ailments.

A Scottish study found that 71% of respondents felt there was no risk with non-prescription medicine (NPM) [22]. Yet, inappropriate SM poses significant danger, sapping medical resources, delaying disease diagnoses, fostering comorbidities, and creating antimicrobial resistance which can cause the dysfunction of medicines [23–25]. Key risks also include drug-drug interactions and adverse drug reactions (ADR) [26], particularly when treatment exceeds durations. Studies from European Union (EU) countries have found that SM significantly increases the risk for ADR, with nearly 1 in 5 patients admitted to the emergency rooms for ADRs having self-medicated via formerly prescribed medications and OTC medications, especially analgesics [27, 28]. There is also a threat of misuse and addiction, which can have devastating effects including deteriorated family relationships, inability to continue employment, loss of children, spouses, and family homes, and potential poisoning, particularly if used to potentiate the effects of alcohol and illicit substances.

Consequently, although appropriate SM is one of the greatest tools for keeping our population healthy, inappropriate SM is a public health issue, and navigating this delicate balance is one of the principal challenges facing health decision-makers in coming years [29]. As such, understanding the state of affairs of SM is vital, and current studies on SM are highly localized, focalized, or fall out of the scope of the EU. Specifically, research oriented toward low-medium income countries or the United States, which experiences higher out-of-pocket drug costs and reduced health care access compared to the EU [20], lacks comparability with the EU. As a result, the objective of this study is, within the scope of the EU, to estimate SM prevalence and examine the association between SM and demographic, socioeconomic, lifestyle, and health factors, overall and by sex.

## Materials and methods

### Data source and study population

This observational study utilizes anonymized data from the third wave of the cross-sectional European Health Interview Surveys (EHIS) [7, 30]. Surveys were conducted between January 2018 and September 2020 on a broad representative sample of the population, consisting of the non-institutionalized subjects aged 15 and over residing in EU countries. Twenty-six of the twenty-seven EU member states are represented in this study, as French data was unavailable via Eurostat. Results are comparable between countries due to the use of a common regulatory framework, including common variable definitions and conceptual guidelines, protocols for survey administration, and a standardized questionnaire, translated as needed to the local population. For homogenization purposes, proxy answers to the dependent variable question were removed. The total weighted sample size for this study is 255,758.

### Study measures

The dependent variable in this study is the dichotomous answer (*yes*/*no*) to the question *“During the past two weeks*,* have you used any medicines or herbal medicines or vitamins not prescribed by a doctor?”* excluding contraceptive pills or hormones used solely for contraception.

Our study also consists of demographic, socioeconomic, lifestyle, and health independent variables. Demographic variables consist of age (separated into five categories: *15–24*, *25–44*, *45–64*, *65–74*, *75+*), sex, marital status and living situation (*single*, *married or living with partner*, *divorced*, or *widowed*), degree of urbanization (*cities*, *towns/suburbs*, or *rural*), and nationality. Nationality has three potential responses: *native-born*, *born in another EU member state*, or *born in a non-EU country*. Due to anonymization practices, Maltese data with respect to nationality was excluded. Socioeconomic variables are also utilized, including education, classified as *no formal education* (below ISCED 1), *primary school* (International Standard Classification of Education [ISCED] 1 or 2), *secondary school* (ISCED 3 or 4), or *higher education* (ISCED 5 or above) [7]. Employment status, categorized as *employed*, *unemployed*, or *inactive* (retirees, students, and those performing domestic tasks, carrying out compulsory service, or unable to work for health reasons) and income level, separated by quintiles within each respective country where the first quintile contains the lowest values and the fifth quintile contains the highest values, comprise the other socioeconomic variables.

Lifestyle variables include smoking (*yes*/*no*), vaping (*yes*/*no*), alcohol consumption (*more than once a month*, *less than once a month*), and physical activity (*low*, *moderate*, *high*) as defined by the methodology used in Jemna et al. 2022 [31]. To measure the impact of health systems on SM, countries are divided into five clusters established by Ferreira et al. 2018 [32]. The clusters are *Austria-Germany*, *Northern countries* (Belgium, Denmark, Finland, Ireland, Luxembourg, Netherlands, and Sweden), *Southern countries* (Cyprus, Greece, Italy, Malta, Portugal, and Spain), *Eastern countries A* (Bulgaria, Hungary, Latvia, Lithuania, Slovakia, and Romania), and *Eastern countries B* (Croatia, Czechia, Estonia, Poland, and Slovenia). It is worth noting that although the clusters are geographically labeled, this is merely for identification purposes, as the clusters are based on health system factors established by the World Health Organization. Specifically, the classifications consider health service provision, generation of health resources, and health financing. Additional health variables used in this study include the presence of a long-standing health problem, use of prescribed medicine in the past two weeks, and visit to a general practitioner/family doctor and visit to medical or surgical specialist in the past 12 months, all measured dichotomously (*yes*/*no*). Finally, four variables evaluating unmet health care needs in the past 12 months with respect to waiting lists, distance or transportation problems, inability to afford medical examination or treatment, and inability to afford prescribed medicines are included, with possible answers including *yes*, *no*, and *no need for health care*.

### Statistical analysis

We calculated the prevalence of SM by country and for each independent variable, selected based on relevancy in the literature, via answers to the dependent variable. For all participant countries, prevalence ratios (PRs) were established to evaluate SM habits compared to the EU total. Furthermore, stratified prevalences for men and women, as well as corresponding odds ratios (OR) with 95% confidence intervals (95%CI) were acquired for all countries and independent variables to evaluate the impact of sex. To estimate the independent effect of the study variables on SM, we calculated the adjusted odds ratios (AOR) and the 95%CI via multivariable logistic regression analysis according to the methodology used by Hosmer et al. [33]. Three models were generated: one for men, a second for women, and a third for both sexes. Estimates were made using the survey command (svy function) in Stata [34], enabling the incorporation of sample design and weights in all statistical calculations. Statistical significance is set as 2-tailed α < 0.05. All figures were created in R using the ggplot2 package [35, 36].

## Results

**Fig. 1:**
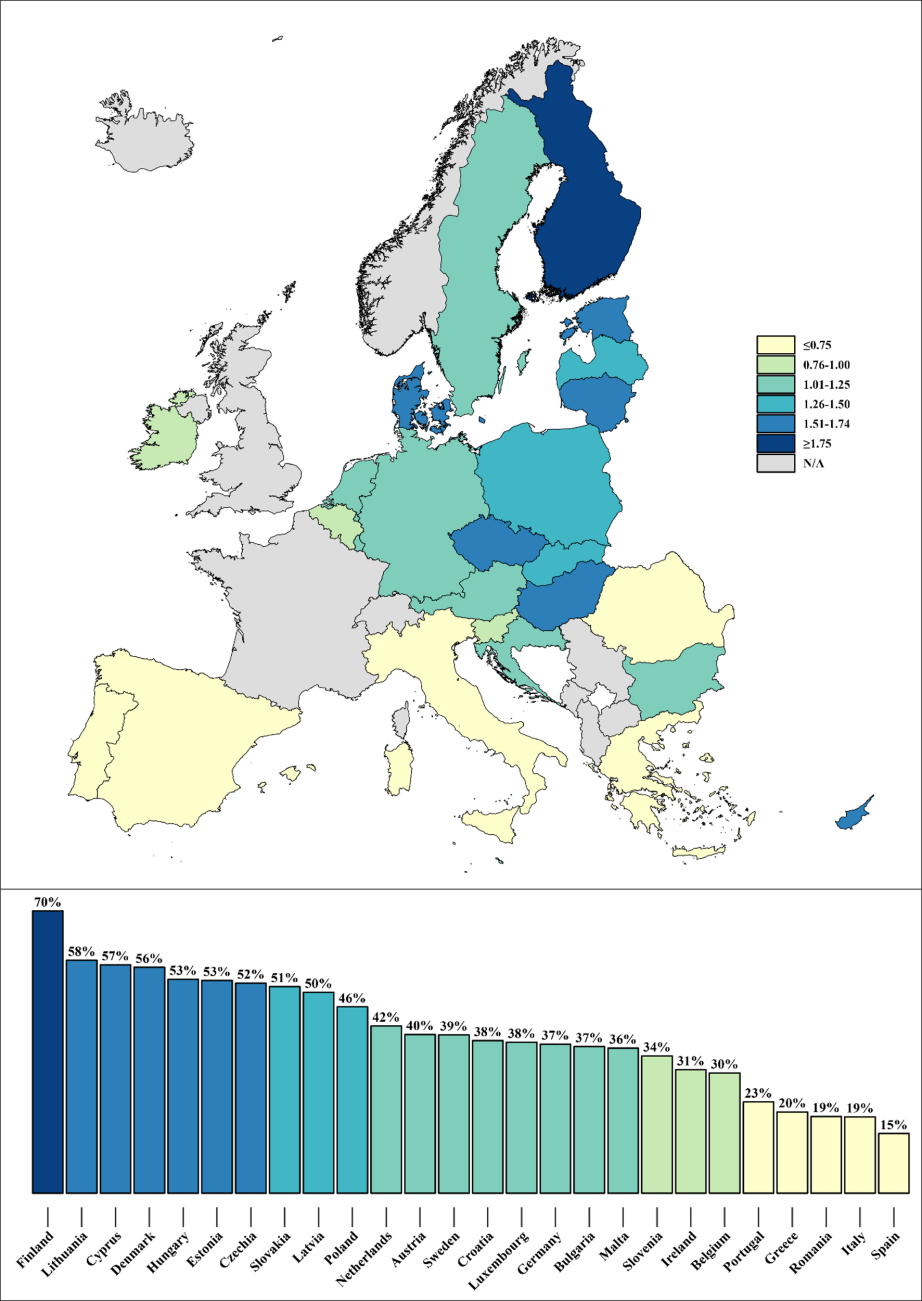
Self-medication prevalence ratios (top) and prevalences (bottom) by country in non-institutionalized residents aged 15 and over in the European Union. European Health Interview Survey Wave 3 (2018–2020)

**Fig. 2:**
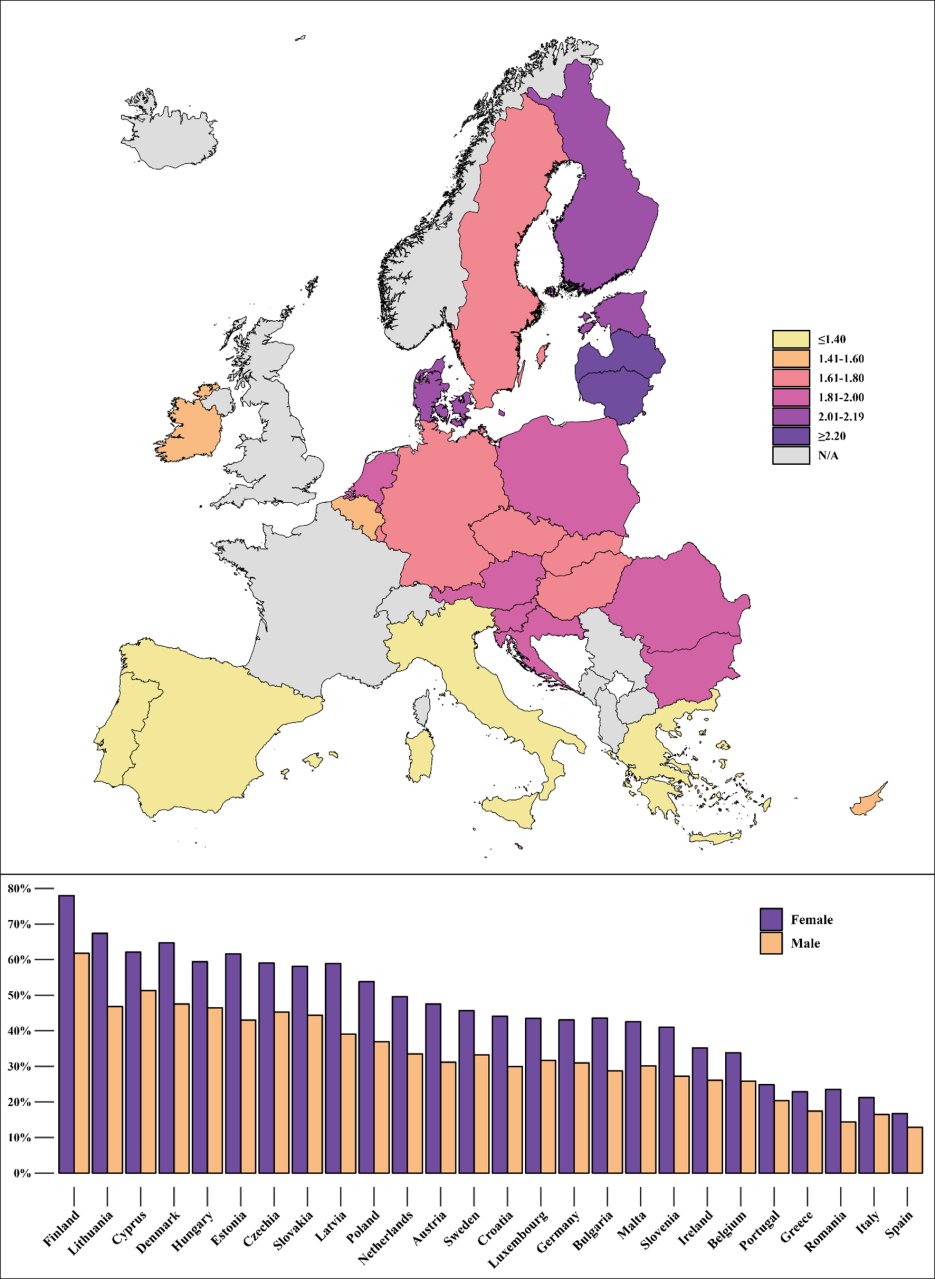
Self-medication female-male odds ratios (top) and female/male prevalences (bottom) by country in non-institutionalized residents aged 15 and over in the European Union. European Health Interview Survey Wave 3 (2018–2020)

In the general EU population, estimated SM prevalence is 34.3% (95%CI = 34.1-34.5%; 91,939/254,746 individuals). SM prevalence by country, as well as the corresponding PRs, ranging from Spain at 0.43 to Finland at 2.05, are displayed in Fig. 1. When separated by sex, estimated SM prevalence is greater in women than in men in all countries, totaling 39.7% (95%CI = 39.5-40.0%) and 28.5% (95%CI = 28.2-28.7%) respectively (OR = 1.65; CI95%=1.60–1.70). These prevalences, as well as the female-male ORs by country, are observable in Fig. 2, where it can be seen that the greatest inequality between female and male SM is found in Lithuania, Latvia, and Finland. The information featured in Figs. 1 and 2 is also available in Supplementary Table A.

The demographic and socioeconomic variable table (Table 1) and lifestyle and health variable table (Table 2) feature SM prevalence in totality and divided by sex, as well as corresponding female-male ORs. The first table highlights that with respect to age, SM prevalence is highest in the 25–44 group (36.1%), and that women display much greater propensity to self-medicate than men in the 45–64 bracket (OR = 1.84; 95%CI = 1.76–1.93). On the other hand, less discrepancy between men and women is observable in the youngest age group, 15–24 (OR = 1.39; 95%CI = 1.26–1.53). Difference in SM prevalence was also registered between those with higher education and those without formal education (39.6% vs. 15.2%), with improved education narrowing the gap between men and women (OR no formal education vs. higher educacation: 1.22 vs. 1.74). Reflected in second table is an inequality that exists between EU health system clusters, with Southern countries experiencing a reported prevalence much below the highest prevalence region, Eastern countries A (17.8% vs. 46.6%). The Southern countries cluster also features a lessened difference between men and women SM prevalence compared to the health system cluster with the largest difference, Eastern Countries B (OR: 1.36 vs. 1.89). SM is also more frequent amongst those who possess a long-standing health problem versus those who do not (38.9% vs. 30.3%).

Table 3 displays the results from the multivariable analysis performed using logistic regression models, partitioned into three models by sex. The model featuring both sexes shows that those between 25 and 44 are most likely to self-medicate (versus ages 75+: AOR = 1.21; 95%CI = 1.12–1.31). Women also exhibit significantly greater propensity to SM (AOR = 1.74; 95%CI = 1.68–1.81). Education displays a strong positive relationship with reported SM, with those having completed higher education being significantly more likely to self-medicate (AOR = 1.83; 95%CI = 1.60–2.09). Being a non-smoker (AOR = 1.05; 95%CI = 1.01–1.10), a vaper (AOR = 1.19; 95%CI = 1.06–1.32), and drinking alcohol more than once a month (AOR = 1.23; 95%CI = 1.19–1.28) is also predictive of SM behavior. By far the strongest indicators for SM across all three models are the health system clusters (Eastern Countries B: AOR = 4.00; 95%CI = 3.81–4.21). Several other health variables, including presence of a long-standing health problem (AOR = 1.39; 95%CI = 1.33–1.45) and visiting a general practitioner or family doctor (AOR = 1.21; 95%CI = 1.15–1.26) or medical or surgical specialist (AOR = 1.21; 95%CI = 1.17–1.26) in the past 12 months serve as conditioners for SM. Having unmet health care needs in the past 12 months due to waiting lists (AOR = 1.38; 95%CI = 1.23–1.55) or inability to afford medical examination or treatment (AOR = 1.27; 95%CI = 1.12–1.42) also significantly contributes to the models.

**Table 1:** Self-medication prevalence by sex in non-institutionalized residents aged 15 and over in the European Union according to demographic and socioeconomic variables. European Health Interview Survey Wave 3 (2018–2020)

		Male		Female		Total		
		*N*	% (95%CI)	*N*	% (95%CI)	*N*	% (95%CI)	OR female (95%CI)
Sex		34,097	28.5 (28.2–28.7)	57,842	39.7 (39.5–40.0)	91,939	34.3 (34.1–34.5)	1.65 (1.60–1.70)
Age	15–24 years	2936	26.7 (26.0-27.3)	4135	33.6 (32.9–34.4)	7071	30.1 (29.6–30.6)	1.39 (1.26–1.53)
	25–44 years	9291	30.5 (30.0-30.9)	14,679	41.8 (41.3–42.2)	23,970	36.1 (35.8–36.4)	1.64 (1.55–1.73)
	45–64 years	11,953	27.8 (27.4–28.2)	21,009	41.5 (41.1–42.0)	32,962	34.8 (34.5–35.1)	1.84 (1.76–1.93)
	65–74 years	5966	28.4 (27.7–29.1)	10,126	39.8 (39.1–40.5)	16,092	34.6 (34.1–35.1)	1.66 (1.56–1.77)
	75 + years	3951	27.0 (26.2–27.8)	7893	35.7 (35.0-36.4)	11,844	32.2 (31.6–32.7)	1.50 (1.38–1.63)
Nationality*	Native-born	30,781	28.5 (28.3–28.8)	52,036	39.9 (39.6–40.2)	82,817	34.4 (34.2–34.6)	1.66 (1.61–1.71)
	Born in another EU state	1315	30.7 (29.2–32.1)	2329	42.2 (40.7–43.6)	3644	36.9 (35.8–37.9)	1.65 (1.51–1.79)
	Born in non-EU country	1909	26.4 (25.4–27.3)	3305	35.9 (34.9–36.9)	5214	31.2 (30.6–31.9)	1.56 (1.37–1.78)
Degree of urbanization	Cities	12,987	29.8 (29.4–30.1)	21,968	40.2 (39.8–40.6)	34,955	35.2 (34.9–35.5)	1.59 (1.52–1.66)
	Towns and suburbs	10,527	28.0 (27.6–28.5)	17,536	39.3 (38.8–39.7)	28,063	33.9 (33.5–34.2)	1.66 (1.58–1.75)
	Rural areas	10,465	27.0 (26.6–27.5)	18,115	39.4 (38.9–39.9)	28,580	33.4 (33.0-33.7)	1.76 (1.67–1.85)
Marital status	Single	7770	27.6 (27.1–28.0)	9814	37.5 (36.9–38.0)	17,584	31.8 (31.5–32.2)	1.57 (1.47–1.68)
	Married or living with partner	22,122	28.9 (28.5–29.2)	32,512	40.2 (39.9–40.6)	54,634	34.6 (34.4–34.9)	1.66 (1.60–1.71)
	Divorced	1617	27.3 (26.1–28.5)	9095	38.2 (37.6–38.9)	10,712	35.8 (35.2–36.4)	1.65 (1.46–1.87)
	Widowed	2490	30.1 (29.1–31.0)	6252	44.4 (43.6–45.3)	8742	38.5 (37.8–39.1)	1.86 (1.67–2.07)
Education level	No formal education	276	13.6 (12.1–15.3)	689	16.1 (14.8–17.6)	965	15.2 (14.1–16.3)	1.22 (1.04–1.62)
	Primary school	6651	20.8 (20.4–21.3)	12,554	30.4 (30.0-30.9)	19,205	26.1 (25.8–26.4)	1.66 (1.55–1.78)
	Secondary school	15,230	30.4 (30.0-30.7)	24,399	42.9 (42.5–43.3)	39,629	36.7 (36.4–36.9)	1.72 (1.65–1.80)
	Higher education	11,704	32.8 (32.3–33.3)	19,867	45.9 (45.4–46.4)	31,571	39.6 (39.2–39.9)	1.74 (1.66–1.81)
Employment status	Employed	18,605	29.4 (29.1–29.7)	26,738	43.2 (42.8–43.6)	45,343	35.8 (35.5–36.0)	1.82 (1.75–1.90)
	Unemployed	1256	21.5 (20.6–22.5)	2130	29.7 (28.6–30.7)	3386	25.6 (24.8–26.3)	1.53 (1.31–1.79)
	Inactive	14,031	28.0 (27.6–28.4)	28,687	37.4 (37.0-37.7)	42,718	33.5 (33.2–33.8)	1.54 (1.47–1.61)
Income level	1st quintile	4549	25.8 (25.2–26.4)	10,353	36.0 (35.4–36.6)	14,902	31.4 (31.0-31.8)	1.62 (1.49–1.76)
	2nd quintile	5622	27.1 (26.5–27.7)	10,676	36.7 (36.1–37.3)	16,298	32.4 (32.0-32.8)	1.56 (1.45–1.67)
	3rd quintile	6674	27.4 (26.9–28.0)	10,995	39.8 (39.2–40.4)	17,669	33.7 (33.3–34.1)	1.74 (1.63–1.86)
	4th quintile	7213	28.7 (28.2–29.3)	10,967	41.0 (40.4–41.6)	18,180	34.9 (34.4–35.3)	1.72 (1.62–1.83)
	5th quintile	7805	31.6 (31.1–32.2)	10,677	43.3 (42.7–43.9)	18,482	37.2 (36.8–37.6)	1.65 (1.56–1.74)

* = does not include data from Malta; OR = odds ratio; CI = confidence interval; EU = European Union; Inactive = retirees, students, and those performing domestic tasks, carrying out compulsory service, or unable to work for health reasons; 1st quintile contains the lowest values and the 5th quintile the highest values

**Table 2:** Self-medication prevalence by sex in non-institutionalized residents aged 15 and over in the European Union according to lifestyle and health variables. European Health Interview Survey Wave 3 (2018–2020)

		Male		Female		Total		
		*N*	% (95%CI)	*N*	% (95%CI)	*N*	% (95%CI)	OR female (95%CI)
Smoking	Yes	8845	28.0 (27.6–28.5)	10,091	40.2 (39.6–40.8)	18,936	33.1 (32.7–33.5)	1.72 (1.62–1.84)
	No	25,034	28.7 (28.4–29.0)	47,400	39.7 (39.4–40.0)	72,434	34.8 (34.6–35.0)	1.63 (1.58–1.69)
Vaping	Yes	1250	34.3 (33.0-35.6)	1266	49.5 (47.7–51.3)	2516	40.0 (39.0-41.1)	1.87 (1.57–2.24)
	No	32,516	28.3 (28.1–28.6)	56,050	39.6 (39.4–39.9)	88,566	34.2 (34.1–34.4)	1.66 (1.61–1.71)
Alcohol consumption	More than once a month	14,868	30.2 (29.8–30.6)	13,075	45.1 (44.5–45.6)	27,943	35.5 (35.2–35.8)	1.89 (1.80–1.99)
	Once a month or less	15,886	27.1 (26.8–27.4)	39,360	38.5 (38.2–38.8)	55,246	34.0 (33.8–34.3)	1.68 (1.61–1.75)
Physical activity	Low physical activity	16,763	26.1 (25.7–26.4)	30,850	36.4 (36.0-36.7)	47,613	31.7 (31.4–31.9)	1.62 (1.56–1.68)
	Moderate physical activity	6660	27.8 (27.2–28.3)	12,397	41.3 (40.7–41.8)	19,057	35.1 (34.7–35.5)	1.83 (1.72–1.94)
	High physical activity	10,592	32.7 (32.3–33.2)	14,414	46.0 (45.5–46.6)	25,006	38.8 (38.5–39.2)	1.75 (1.65–1.86)
Health system cluster	Austria-Germany	5685	31.0 (30.5–31.5)	9432	43.5 (43.0–44.0)	15,117	37.4 (37.0-37.7)	1.71 (1.59–1.84)
	Northern countries	8249	35.4 (34.8–36.1)	13,248	48.9 (48.2–49.6)	21,497	42.3 (41.8–42.7)	1.74 (1.67–1.82)
	Southern countries	6915	15.5 (15.1–15.9)	10,293	20.0 (19.6–20.4)	17,208	17.8 (17.5–18.1)	1.36 (1.29–1.44)
	Eastern countries A	6339	29.5 (28.9–30.2)	11,564	42.4 (41.7–43.1)	17,903	36.3 (35.8–36.8)	1.75 (1.67–1.84)
	Eastern countries B	6909	38.0 (37.3–38.7)	13,305	53.7 (53.0-54.4)	20,214	46.6 (46.1–47.1)	1.89 (1.78–1.99)
Long-standing health problem	Yes	18,259	32.6 (32.2–33.0)	33,454	44.0 (43.6–44.3)	51,713	38.9 (38.7–39.2)	1.62 (1.56–1.69)
	No	15,657	25.3 (25.0-25.6)	24,101	35.4 (35.1–35.8)	39,758	30.2 (30.0-30.5)	1.62 (1.55–1.68)
Use of prescribed medicine	Yes	18,255	30.8 (30.4–31.2)	34,848	42.3 (41.9–42.6)	53,103	37.3 (37.1–37.6)	1.65 (1.58–1.71)
	No	15,800	26.6 (26.3–27.0)	22,920	36.8 (36.4–37.1)	38,720	31.4 (31.2–31.7)	1.60 (1.54–1.67)
Visit to a general practitioner or family doctor in the past 12 months	Yes	25,929	30.1 (29.8–30.4)	47,304	41.0 (40.7–41.3)	73,233	36.1 (35.9–36.3)	1.61 (1.56–1.67)
	No	8089	24.3 (23.9–24.8)	10,420	34.7 (34.1–35.2)	18,509	28.9 (28.5–29.2)	1.65 (1.56–1.75)
Visit to a medical or surgical specialist in the past 12 months	Yes	17,635	32.1 (31.8–32.5)	35,477	43.2 (42.8–43.5)	53,112	38.6 (38.3–38.8)	1.60 (1.54–1.67)
	No	16,315	25.5 (25.2–25.9)	22,128	35.0 (34.6–35.4)	38,443	29.8 (29.6–30.1)	1.57 (1.51–1.64)
Unmet need for health care due to waiting lists in the past 12 months	Yes	5244	37.9 (37.1–38.7)	10,986	49.5 (48.8–50.1)	16,230	44.7 (44.2–45.2)	1.60 (1.48–1.73)
	No	21,099	28.0 (27.6–28.3)	36,277	38.1 (37.8–38.4)	57,376	33.3 (33.1–33.6)	1.59 (1.53–1.64)
	No need for health care	7616	25.5 (25.1–26.0)	10,357	37.2 (36.7–37.8)	17,973	30.7 (30.4–31.1)	1.73 (1.62–1.84)
Unmet need for health care due to distance or transportation problems in the past 12 months	Yes	1010	42.0 (40.3–43.8)	2208	48.3 (46.8–49.7)	3218	45.8 (44.6–46.9)	1.23 (1.05–1.58)
	No	25,161	29.1 (28.8–29.3)	44,643	39.8 (39.5–40.1)	69,804	34.9 (34.7–35.1)	1.62 (1.56–1.67)
	No need for health care	7757	25.7 (25.3–26.2)	10,724	38.0 (37.4–38.6)	18,481	31.2 (30.8–31.6)	1.77 (1.66–1.88)
Unmet need for health due to inability to afford medical examination or treatment in the past 12 months	Yes	1578	40.6 (39.0-42.1)	3624	50.1 (48.9–51.3)	5202	46.5 (45.6–47.5)	1.47 (1.27–1.71)
	No	23,420	28.7 (28.4–29.0)	41,423	39.5 (39.2–39.8)	64,843	34.5 (34.3–34.7)	1.62 (1.57–1.68)
	No need for health care	7897	26.7 (26.3–27.2)	10,922	38.8 (38.2–39.3)	18,819	32.2 (31.9–32.6)	1.73 (1.63–1.84)
Unmet need for health care due to inability to afford prescribed medicines in the past 12 months	Yes	1279	37.3 (35.7–38.9)	2858	48.3 (46.9–49.6)	4137	43.8 (42.8–44.9)	1.57 (1.32–1.85)
	No	23,643	29.1 (28.8–29.4)	42,438	40.1 (39.8–40.4)	66,081	35.0 (34.8–35.2)	1.63 (1.57–1.68)
	No need for health care	7940	26.1 (25.7–26.6)	10,631	37.7 (37.1–38.3)	18,571	31.3 (30.9–31.7)	1.71 (1.61–1.82)

OR = odds ratio; CI = confidence interval; Northern countries = Belgium, Denmark, Finland, Ireland, Luxembourg, Netherlands, and Sweden; Southern countries = Cyprus, Greece, Italy, Malta, Portugal, and Spain; Eastern Countries A = Bulgaria, Hungary, Latvia, Lithuania, Slovakia, and Romania; Eastern Countries B = Croatia, Czechia, Estonia, Poland, and Slovenia

**Table 3:** Multivariable regression analysis of factors associated with self-medication by sex in non-institutionalized residents aged 15 and over in the European Union. European Health Interview Survey Wave 3 (2018–2020)

		Male	Female	Total
		AOR (95%CI)	AOR (95%CI)	AOR (95%CI)
Age	75+	1.00	1.00	1.00
	15–24	1.29 (1.11–1.49)	1.06 (0.93–1.20)	1.16 (1.06–1.28)
	25–44	1.22 (1.08–1.38)	1.21 (1.09–1.34)	1.21 (1.12–1.31)
	45–64	1.04 (0.93–1.16)	1.18 (1.07–1.29)	1.11 (1.03–1.19)
	65–74	0.98 (0.89–1.08)	1.07 (0.99–1.17)	1.03 (0.97–1.10)
Sex	Male			1.00
	Female			1.74 (1.68–1.81)
Nationality*	Native-born	1.00	1.00	1.00
	Born in another EU member state	1.15 (0.98–1.36)	1.18 (1.02–1.35)	1.16 (1.04–1.30)
	Born in non-EU country	1.04 (0.92–1.18)	1.07 (0.96–1.19)	1.05 (0.97–1.14)
Degree of urbanization	Rural areas	1.00	1.00	1.00
	Cities	1.19 (1.11–1.26)	1.09 (1.03–1.15)	1.14 (1.09–1.19)
	Towns and suburbs	1.07 (1.00-1.15)	1.02 (0.96–1.08)	1.05 (1.00-1.09)
Education level	No formal education	1.00	1.00	1.00
	Primary school	1.14 (0.91–1.42)	1.26 (1.08–1.48)	1.25 (1.10–1.42)
	Secondary education	1.48 (1.18–1.84)	1.58 (1.35–1.86)	1.58 (1.38–1.80)
	Higher education	1.70 (1.36–2.12)	1.86 (1.58–2.20)	1.83 (1.60–2.09)
Employment status	Unemployed	1.00	1.00	1.00
	Employed	1.16 (1.01–1.36)	1.29 (1.14–1.47)	1.24 (1.12–1.37)
	Inactive	1.15 (1.00-1.36)	1.21 (1.06–1.38)	1.18 (1.06–1.31)
Income level	1st quintile	1.00	1.00	1.00
	2nd quintile	1.08 (0.98–1.19)	1.04 (0.97–1.13)	1.06 (0.99–1.12)
	3rd quintile	1.07 (0.97–1.17)	1.11 (1.03–1.20)	1.09 (1.03–1.16)
	4th quintile	1.11 (1.01–1.22)	1.14 (1.06–1.23)	1.13 (1.06–1.19)
	5th quintile	1.16 (1.06–1.27)	1.12 (1.03–1.21)	1.14 (1.07–1.21)
Smoking	Yes	1.00	1.00	1.00
	No	1.04 (1.01–1.13)	1.07 (1.00-1.14)	1.05 (1.01–1.10)
Vaping	No	1.00	1.00	1.00
	Yes	1.14 (1.03–1.31)	1.23 (1.03–1.47)	1.19 (1.06–1.32)
Alcohol consumption	Less than once a month	1.00	1.00	1.00
	More than once a month	1.19 (1.13–1.26)	1.30 (1.23–1.38)	1.23 (1.19–1.28)
Physical activity	Low physical activity	1.00	1.00	1.00
	Moderate physical activity	1.06 (0.99–1.14)	1.08 (1.02–1.14)	1.08 (1.03–1.12)
	High physical activity	1.29 (1.21–1.37)	1.22 (1.15–1.29)	1.27 (1.22–1.32)
Health system cluster	Southern countries	1.00	1.00	1.00
	Austria-Germany	2.17 (2.00-2.36)	2.80 (2.60–3.01)	2.51 (2.37–2.65)
	Northern countries	2.83 (2.63–3.04)	3.69 (3.45–3.94)	3.29 (3.13–3.45)
	Eastern countries A	2.61 (2.43–2.80)	3.52 (3.31–3.74)	3.09 (2.95–3.23)
	Eastern countries B	3.30 (3.06–3.57)	4.65 (4.35–4.96)	4.00 (3.81–4.21)
Long-standing health problem	No	1.00	1.00	1.00
	Yes	1.38 (1.29–1.47)	1.41 (1.33–1.49)	1.39 (1.33–1.45)
Visit to a general practitioner or family doctor in the past 12 months	No	1.00	1.00	1.00
	Yes	1.23 (1.15–1.32)	1.18 (1.11–1.25)	1.21 (1.15–1.26)
Visit to a medical or surgical specialist in the past 12 months	No	1.00	1.00	1.00
	Yes	1.18 (1.11–1.26)	1.24 (1.18–1.31)	1.21 (1.17–1.26)
Unmet need for health care due to waiting lists in the past 12 months	No need for health care	1.00	1.00	1.00
	Yes	1.32 (1.1–1.58)	1.43 (1.24–1.65)	1.38 (1.23–1.55)
	No	1.01 (0.86–1.2)	1.07 (0.94–1.22)	1.04 (0.94–1.16)
Unmet need for health care due to inability to afford medical examination or treatment in the past 12 months	No need for health care	1.00	1.00	1.00
	Yes	1.38 (1.13–1.69)	1.20 (1.03–1.38)	1.27 (1.12–1.42)
	No	0.99 (0.90–1.08)	0.98 (0.90–1.06)	0.98 (0.92–1.04)

* = does not include data from Malta; AOR = adjusted odds ratio; CI = confidence interval; EU = European Union; Inactive = retirees, students, and those performing domestic tasks, carrying out compulsory service, or unable to work for health reasons; 1st quintile contains the lowest values and the 5th quintile the highest values; Southern countries = Cyprus, Greece, Italy, Malta, Portugal, and Spain; Northern countries = Belgium, Denmark, Finland, Ireland, Luxembourg, Netherlands, and Sweden; Eastern Countries A = Bulgaria, Hungary, Latvia, Lithuania, Slovakia, and Romania; Eastern Countries B = Croatia, Czechia, Estonia, Poland, and Slovenia

## Discussion

To the authors´ knowledge, this study is the first of this scale to identify SM prevalence and associated factors in the general EU population. SM prevalence varies significantly across scientific literature based on population characteristics and methodological differences. Nonetheless, the estimated prevalence found in our study, 34.3% during a two-week sample window, is comparable with previous studies featuring similar characteristics. A systematic review performed during the COVID-19 pandemic discovered that European SM prevalence was the lowest of all geographical regions at 40.8% [37], potentially attributable to greater access to prescribed medications [38]. In Germany, one 2015 study reported a seven-day OTC consumption prevalence of 46.3% [39], while a 2017 study reporting on the second wave of the EHIS found a two-week self-medication prevalence of 42.1% [40]. Similarly, a study performed in Paris, France in 2018 placed SM prevalence during the past four weeks at 53.5% [41]. In our study, prevalence varied substantially between countries.

Along the same lines, our multivariable analysis concludes that health clusters are the strongest determinant of SM in the EU. We discover that residents of Eastern Countries B are four times as likely to self-medicate (AOR = 4.00; 95%CI = 3.81–4.21) with respect to those of Southern countries. Previous research working with data from the first iteration of the EHIS found similar results among the elderly, noting the highest SM prevalence in Poland, part of Eastern Countries B cluster, and the lowest in Spain, a member of the Southern countries cluster [42]. Additional research also shows that Poland and Czechia, members of the Eastern Countries B block, display extremely high rates of SM at 59% and 85% respectively [5, 43]. This result emphasizes the substantial influence of localized EU health systems on population health behaviors. Specifically, health service provision, generation of health resources, and health financing clearly have a substantial impact on SM, even surpassing the impact of traditional socioeconomic, demographic, health, or lifestyle variables. As such, it highlights the need to reconsider traditional epidemiological determinants and underscores the importance of health systems in shaping our health practices. It is also worth noting that across the board, women are more significantly influenced to self-medicate by their country´s health system than men, providing yet another example of how the gender influences health.

In addition, our study identifies young adults (ages 25–44) as the most likely to self-medicate (AOR = 1.21; 95%CI = 1.12–1.31). These findings are congruent with prior research that found that SM is highest at younger ages and trends downward as age increases [4, 40, 44, 45]. Younger people tend to seek less medical attention, face less comorbidities, and perceive their health as better than those who are older [46, 47], which may make SM an appealing option versus prescription medications. A cross-sectional study in the United Kingdom highlighted that younger age is associated with increased risk for misuse of non-prescription medications, which also could contribute to SM [48]. Similarly, an Australian study by Vong et al. also found that younger individuals are less likely to follow directions for non-prescription medicines [6]. Coupled with the fact that younger people tend to care less about and seek less health information [49], these findings highlight the importance of finding new ways to educate young people about safe SM.

Consistent with findings from previous studies [5, 16, 39, 40, 45, 50, 51], our study shows that women are more likely to self-medicate than men (AOR = 1.74; 95%CI = 1.68–1.81). This finding is consistent across the EU, though the difference between men and women varies across countries. Research has cited barriers to health care, such as unmet needs stemming from long wait times [52] or socioeconomic disparities related to occupation, income, and education [53] as explanations for women´s greater propensity self-medicate. However, while these could be contributing factors, we find that the relationship between sex and SM continues even in multivariable models separating these variables, indicating that these are not the primary driving forces of this inequality. Instead, we find more convincing explanations surrounding women´s greater inclination toward self-care [42, 49]. A Finnish study by Ek on gender differences in health information highlighted that women are more engaged and informed health decision-makers, more frequently seeking health information from lay sources and displaying interest in health repercussions [49]. In the same vein, gender bias in the treatment of conditions and prescription of medicines is well-documented [54], and women could turn to SM to ease the burden of certain ailments. Men, on the other hand, are often encouraged to “tough it out” and avoid medication [55]. This contrast underscores the multidimensional nature of gender roles and the ways in which they manifest themselves in health habits. Regardless, in the variables collected in this study, the factors influencing SM in men and women are highly comparable.

Our study also reveals that immigrants born in other EU states have an increased likelihood to self-medicate (AOR = 1.16; 95%CI = 1.04–1.30) compared to native-born individuals. These findings align with previous research conducted in Spain which also found immigrant status to be a factor associated with SM [45]. Immigrants may lack the same supportive familiar system or access to health care as native-born residents, making SM an appealing tool. Alternatively, those with the resources to move to other EU member states may be particularly equipped with greater self-care consciousness. Notably, this finding was not statistically significant in those born outside the EU, which may be attributable to a lack of familiarity with or access to EU health services.

In line with previous research [51, 56], our study shows that residing in cities (AOR = 1.14; 95%CI = 1.09–1.19) is associated with an increased likelihood of self-medicating in comparison with rural living. This result may be attributable to greater access in urban areas to pharmacies or other stores selling SM products, or greater specialist use in cities versus general practitioners in rural areas, wherein responsibility for smaller health issues falls more upon the individual [56]. Pollution or other consequences of urban living could also be contributing factors, as a study by Davies et al. discovered a positive relationship between OTC medication sales and environmental contaminants found in cities [57].

In our multivariable model that encompasses both sexes, individuals who have completed higher education are 83% more likely to engage in SM than those without any formal education (AOR = 1.83; CI95%=1.60–2.09). This positive relationship is incremental between all levels of SM and education, as is common in health indicators [47]. These findings align with previous research, as a number of studies have identified a positive correlation between these two variables across diverse demographic groups [4, 5, 16, 40, 42, 44, 45, 51, 58]. More educated individuals may self-medicate more given their generally greater health literacy [59] and may consequently engage in greater self-care. Education level is also positively associated with social networking, which could lead to medication sharing and lay recommendations [52]. Concerningly, however, in an antibiotic study, more appropriate consumption behaviors were found in subjects with lower education levels, despite the fact that they were less health literate, because those with higher education were more likely to question their doctor´s orders [60]. Similar findings were yielded by Vong et al. in Australia who discovered reduced likelihood amongst the most educated to follow directions on non-prescription medicines [6]. In other words, education may grant excessive confidence in patient decision-making with respect to medications. This phenomenon may be specific to developed countries or the sphere of the EU, where a higher baseline level of health literacy and health care access exists, as a systematic review of antibiotic consumption found that while worldwide, improved education level is associated with significantly lower odds of misuse, in Europe, the opposite is true [59].

With respect to income, our findings align with existing literature: while a relationship exists between greater income and SM (AOR = 1.14; 95%CI = 1.07–1.21), the robust association often cited is exclusive to bivariate analysis, falling apart in multivariable analysis. On its surface, this result may appear surprising, as increased income improves access to non-reimbursed medicine [41]. Instead, we find that this relationship is mediated by other variables, notably education, employment status (AOR = 1.24; 95%CI = 1.12–1.37) and barriers to health care, including unmet needs due to inability to afford medical examination or treatment (AOR = 1.27; 95%CI = 1.12–1.42) and waiting lists (AOR = 1.38; 95%CI = 1.23–1.55). The latter has been identified as a substantial and common barrier to health care utilization [52, 53] as greater income permits the affordance of superior private health care plans, bypassing the public system waiting lists [41]. Put shortly, income becomes a more relevant factor when it becomes a barrier to accessing medical care. Our findings also suggest that women may be more likely to self-medicate in response to waiting lists (AOR: 1.43 vs. 1.32) while men may be motivated to self-medicate due to inability to afford medical examination or treatment (AOR: 1.38 vs. 1.20). A possible explanation is that waiting lists may be a less traversable barrier than inability to afford medical examination or treatment.

In the same vein, our results reveal that visits to general practitioners and family doctors (AOR = 1.21; 95%CI = 1.15–1.26), as well as medical or surgical specialists (AOR = 1.21; 95%CI = 1.17–1.26), are positively associated with SM. These findings are likely attributable to greater prophylaxis among those who receive regular medical care. Though some previous studies have logically linked lack of doctor visits to SM [45], this relationship may only be applicable where SM serves as a substitute for treatment by a medical professional due to health care barriers. Our study also finds that presence of a long-standing health problem serves as a risk factor for SM (AOR = 1.39; 95%CI = 1.33–1.45). Findings on the effects of chronic conditions on SM are mixed, as some subjects may have regular medical treatment and rely on prescription medications to manage their ailments, while others engage in SM due to experience in dealing with their illnesses [3, 41, 42, 45, 55, 61, 62]. A study performed in the greater Paris area by Vanhaesebrouck et al. failed to find a relationship between the two variables due to this phenomenon [41]. A UK general population study revealed that use of NPM with a long-standing illness is predictive of NPM abuse [48], making this risk factor particularly significant as it may reflect inappropriate SM.

Concerning lifestyle habits, alcohol consumption (AOR = 1.23; 95%CI = 1.19–1.28), smoking (AOR = 1.05; 95%CI = 1.01–1.10), and vaping (AOR = 1.19; 95%CI = 1.06–1.32) display positive associations with SM. Previous studies have linked alcohol use [42, 45] and vaping [63] to SM. With respect to smoking, an association with SM has been reported in women (but not men) [45], in adolescents [64], and for OTC analgesic use [3], while this study highlights a broader connection. Both smoking and alcohol use have ironically been found to be positively associated with self-perceived health, as ill individuals may abstain from such practices [46]. The same could be applicable to SM, as those capable of engaging in smoking, vaping, and alcohol drinking may be healthier individuals. Alternatively, SM may be more frequent for these individuals in reaction to the negative health consequences of said habits. It is also possible that this relationship is explained by overlapping motivators between SM and these habits, which in and of themselves could be considered ways of self-medicating. Additionally, our study reveals that greater physical activity also emerges as a factor associated with SM (AOR = 1.27; 95%CI = 1.22–1.32). This may reflect also self-care habits, as the employed concept of SM encapsulates more than non-prescription medicine, including products like vitamins and supplements, which are often favored by those who are physically active. A study of French adults using the same definition of SM also found a positive association between SM and physical activity, though the results were non-significant [31].

This study is subject to a series of limitations. The first limitation stems from the cross-sectional nature of the data, which does not allow us to establish causality. Second, the EHIS did not collect data regarding consumption of specific medication classes, which may display differing relationships with the variables. Future studies including this component could help to disentangle the heterogeneity of SM. Along the same lines, the EHIS made reference to binary sex, which may inadequately capture the range of diversity and social, political, and economic forces expressed in gender [65]. Furthermore, our study features an uneven sample size between countries, with several countries with low SM prevalences having amongst the largest sample sizes, such as Spain and Italy, which could have led to an underestimation of SM prevalence in the EU. Additionally, social desirability bias also could have led to underreporting on SM. This is furthered by the fact that several differences existed between countries in data collection methods and sampling design, which could have altered results and reduce comparability between countries [66]. In the case of this study, individuals interviewed face-to-face or over the phone may have felt additional social pressure to underreport self-medicating versus those who filled out self-administered written or online surveys.

Moreover, data was collected non-simultaneously across the EU, as surveys were carried out over multiple years and seasons, and consequently, seasonal and annual variance of conditions that provoke SM could have been impactful. Specifically, lower temperatures are associated with greater OTC respiratory medication sales [67], and as a result, SM prevalence could be greater in colder months. It is worth noting that the vast majority of responses were collected in autumn. Along the same lines, three countries (Germany, Spain, and Malta) collected data after the beginning of the COVID-19 pandemic, which could alter SM prevalence in either direction [68]. An additional limitation is that the self-reported data contained in this study is subject to recall bias. The two-week window of the dependent variable question should have reduced some of the bidirectional effects of recall bias, but can complicate SM prevalence comparisons with other studies. A German study pointed out that the short reference period and self-assessment method used in the EHIS may lead to prevalence estimates that differ substantially from epidemiological studies [47]. Finally, the non-response rate, ranging from 12 to 78% based on country [68], may also be influential, as those who refused to participate could have shared insights into SM, even if the direction of this effect is indeterminable.

Nonetheless, the quality report of the EHIS 3 details that the data underwent validation, calibration, and non-response adjustments procedures to minimize the effect of all potential sources of sampling and non-sampling errors, resulting in dataset that is highly harmonized and allows for a high degree of comparability across EU member states [68]. Coupled with the robust weighted sample size of 255,758, the authors feel that none of the aforementioned limitations should dampen the strength or relevancy of the findings and their applicability to the EU general population.

## Conclusion

In conclusion, this study reveals that the estimated prevalence of SM in the EU during a two-week period is 34.3%. Our findings demonstrate that while women self-medicate more than men (39.7% vs. 28.5%), the factors that influence the consumption patterns of women and men are very similar. We also identify numerous other factors associated with SM, including youth, higher education, urban living, smoking, vaping, and alcohol consumption. Additional associations include the presence of a long-standing health problem, visits to doctors, and unmet needs for health care. Undoubtedly, however, the strongest conditioners of SM are health systems, reflecting the powerful impact that health care organizational structure has on population behaviors and consequent health outcomes.

We feel that the results of this study can serve as a reference point for future monitoring of SM prevalence, especially given that surveys were administered just prior to the COVID-19 pandemic. We also believe that that the findings of this study can inform targeted public health campaigns to at-risk groups for harmful SM, serve as a baseline for the development of tools to identify potentially dangerous SM practices, and inform future health policy decisions surrounding SM.

## Electronic supplementary material

Below is the link to the electronic supplementary material.

Supplementary Material 1

## Data Availability

Data requests must be filed with Eurostat.

## Code availability

The code for data processing is available upon request.
