# Supplementary material for: Self-medication among general population in the European Union: prevalence and associated factors
Source: Eur J Epidemiol. 2024 Sep 18;39(9):977–90. doi: 10.1007/s10654-024-01153-1 (PMC11470884; doi:10.1007/s10654-024-01153-1)
Supplement: Supplementary file 1 — Supplementary Material 1 [file 10654_2024_1153_MOESM1_ESM.docx]

**Supplementary Table A. Self-medication prevalence by country and sex in non-institutionalized residents aged 15 and over in the European Union. European Health Interview Survey Wave 3 (2018–2020)**

|  | **Male** | | **Female** | | **Total** | |  |  |
| --- | --- | --- | --- | --- | --- | --- | --- | --- |
|  | **N** | **% (95%CI)** | **N** | **% (95%CI)** | **N** | **% (95%CI)** | **PR (Total)** | **OR Female (95%CI)** |
| **Finland** | 1421 | 61.8 (59.6–63.9) | 2546 | 78.0 (76.2–79.7) | 3967 | 70.3 (68.9–71.7) | 2.05 (2.02–2.08) | 2.20 (1.93–2.49) |
| **Lithuania** | 948 | 46.8 (43.8–49.8) | 1965 | 67.4 (64.8–69.9) | 2913 | 58.0 (56.0–60.0) | 1.69 (1.64–1.74) | 2.35 (2.08–2.65) |
| **Cyprus** | 1468 | 51.3 (46.0-56.6) | 1998 | 62.1 (57.0-66.9) | 3466 | 56.9 (53.2–60.5) | 1.66 (1.56–1.75) | 1.56 (1.39–1.74) |
| **Denmark** | 1350 | 47.5 (45.5–49.6) | 2383 | 64.7 (62.8–66.6) | 3733 | 56.3 (54.8–57.7) | 1.64 (1.61–1.67) | 2.02 (1.82–2.25) |
| **Hungary** | 1181 | 46.4 (44.9–48.0) | 1788 | 59.4 (57.9–60.9) | 2969 | 53.3 (52.2–54.4) | 1.55 (1.53–1.58) | 1.69 (1.51–1.89) |
| **Estonia** | 867 | 43.0 (38.9–47.5) | 1758 | 61.6 (57.5–65.4) | 2625 | 53.0 (50.0–56.0) | 1.54 (1.46–1.62) | 2.12 (1.88–2.40) |
| **Czechia** | 1570 | 45.2 (43.7–46.7) | 2682 | 59.0 (57.6–60.5) | 4252 | 52.3 (51.3–53.4) | 1.52 (1.50–1.55) | 1.74 (1.58–1.93) |
| **Slovakia** | 1056 | 44.4 (42.3–46.5) | 1950 | 58.1 (56.1–60.1) | 3006 | 51.5 (50.0-52.9) | 1.50 (1.46–1.53) | 1.74 (1.54–1.95) |
| **Latvia** | 934 | 39.1 (35.5–42.8) | 2023 | 58.9 (55.5–62.1) | 2957 | 50.0 (47.5–52.5) | 1.46 (1.39–1.52) | 2.23 (1.99–2.51) |
| **Poland** | 2578 | 36.9 (36.0-37.8) | 5353 | 53.8 (53.0-54.6) | 7931 | 46.4 (45.8–47.0) | 1.35 (1.34–1.36) | 1.99 (1.84–2.15) |
| **Netherlands** | 1328 | 33.5 (32.4–34.6) | 2077 | 49.6 (48.5–50.8) | 3405 | 41.6 (40.8–42.4) | 1.21 (1.20–1.23) | 1.95 (1.78–2.14) |
| **Austria** | 2179 | 31.2 (29.7–32.7) | 3908 | 47.5 (45.9–49.1) | 6087 | 39.5 (38.4–40.6) | 1.15 (1.12–1.18) | 1.99 (1.84–2.16) |
| **Sweden** | 1620 | 33.2 (31.8–34.6) | 2196 | 45.6 (44.1–47.2) | 3816 | 39.4 (38.4–40.5) | 1.15 (1.12–1.17) | 1.69 (1.55–1.84) |
| **Croatia** | 665 | 30.0 (27.6–32.4) | 1241 | 44.1 (41.8–46.3) | 1906 | 38.0 (36.4–39.7) | 1.11 (1.06–1.15) | 1.84 (1.58–2.14) |
| **Luxembourg** | 632 | 31.7 (26.2–37.7) | 1035 | 43.5 (37.6–49.7) | 1667 | 37.6 (33.5–42.0) | 1.10 (0.98–1.22) | 1.66 (1.46–1.89) |
| **Germany** | 3506 | 31.0 (30.5–31.5) | 5524 | 43.1 (42.5–43.6) | 9030 | 37.1 (36.8–37.5) | 1.08 (1.08–1.09) | 1.68 (1.55–1.83) |
| **Bulgaria** | 984 | 28.7 (27.0-30.4) | 1742 | 43.6 (41.8–45.4) | 2726 | 36.5 (35.3–37.8) | 1.06 (1.03–1.09) | 1.92 (1.73–2.13) |
| **Malta** | 604 | 30.2 (24.4–36.5) | 968 | 42.5 (36.2–49.6) | 1572 | 36.1 (31.8–40.8) | 1.05 (0.93–1.18) | 1.71 (1.49–1.96) |
| **Slovenia** | 1229 | 27.3 (24.4–30.4) | 2271 | 41.0 (37.8–44.3) | 3500 | 34.2 (32.0-36.4) | 1.00 (0.94–1.06) | 1.85 (1.69–2.03) |
| **Ireland** | 865 | 26.1 (24.2–28.1) | 1432 | 35.2 (33.2–37.4) | 2297 | 30.8 (29.3–32.2) | 0.90 (0.86–0.93) | 1.54 (1.35–1.74) |
| **Belgium** | 1033 | 25.8 (24.5–27.2) | 1579 | 33.8 (32.4–35.3) | 2612 | 30.0 (29.0–31.0) | 0.87 (0.85–0.90) | 1.47 (1.29–1.67) |
| **Portugal** | 1214 | 20.3 (19.1–21.6) | 2063 | 24.8 (23.6–26.1) | 3277 | 22.7 (21.9–23.6) | 0.66 (0.64–0.68) | 1.29 (1.14–1.47) |
| **Greece** | 674 | 17.4 (16.3–18.6) | 1013 | 22.9 (21.6–24.1) | 1687 | 20.2 (19.4–21.1) | 0.59 (0.57–0.61) | 1.40 (1.23–1.60) |
| **Romania** | 1236 | 14.4 (13.6–15.2) | 2096 | 23.5 (22.6–24.4) | 3332 | 19.1 (18.5–19.7) | 0.56 (0.54–0.57) | 1.83 (1.66–2.02) |
| **Italy** | 1696 | 16.5 (15.5–17.4) | 2479 | 21.3 (20.3–22.2) | 4175 | 19.0 (18.3–19.7) | 0.55 (0.54–0.57) | 1.37 (1.27–1.48) |
| **Spain** | 1259 | 12.9 (12.4–13.4) | 1772 | 16.8 (16.2–17.3) | 3031 | 14.9 (14.5–15.2) | 0.43 (0.42–0.44) | 1.36 (1.24–1.50) |
| **TOTAL** | 34097 | 28.5 (28.2–28.7) | 57842 | 39.7 (39.5–40.0) | 91939 | 34.3 (34.2–34.5) | 1.00 (1.00–1.00) | 1.65 (1.60–1.70) |

PR = prevalence ratio; OR = odds ratio; CI = confidence interval
